# Low carbon futures: assessing the status of decarbonisation efforts at universities within a 2050 perspective

**DOI:** 10.1186/s13705-023-00384-6

**Published:** 2023-02-16

**Authors:** Walter Leal Filho, Diogo Guedes Vidal, Maria Alzira Pimenta Dinis, Wim Lambrechts, Claudio R. P. Vasconcelos, Petra Molthan-Hill, Ismaila Rimi Abubakar, Rachel M. Dunk, Amanda Lange Salvia, Ayyoob Sharifi

**Affiliations:** 1 European School of Sustainability Science and Research, Hamburg University of Applied Sciences, Ulmenliet 20, 21033 Hamburg, Germany; 2 Department of Natural Sciences, Manchester Metropolitan University, Chester Street, Manchester, M11 5GD UK; 3 Department of Life Sciences, Faculty of Sciences and Technology, Center for Functional Ecology-Science for People and The Planet (CFE), TERRA Associate Laboratory, University of Coimbra, Calçada Martim de Freitas, 3000-456 Coimbra, Portugal; 4 UFP Energy, Environment and Health Research Unit (FP-ENAS), University Fernando Pessoa (UFP), Praça 9 de Abril 349, 4249-004 Porto, Portugal; 5 Department of Marketing and Supply Chain Management, Faculty of Management, Open Universiteit, The Netherlands (OUNL), Postbus 2960, 6401 DL Heerlen, The Netherlands; 6 Laboratory of Sustainability Engineering and Consumption, Federal University of Paraíba, João Pessoa, PB Brazil; 7 Algoritmi Research Centre, School of Engineering, University of Minho, 4800-058 Guimarães, Portugal; 8 Business Sustainability, Nottingham Trent University, England, UK; 9 College of Architecture and Planning, Imam Abdulrahman Bin Faisal University (Formerly, University of Dammam), P.O. Box 1982, Dammam, 31441 Saudi Arabia; 10 School of Science and the Environment, Manchester Metropolitan University, Chester Street, Manchester, M1 5GD UK; 11 Graduate Program in Civil and Environment Engineering, University of Passo Fundo, Campus I-BR 285, São José, Passo Fundo, RS 99052-900 Brazil; 12 Graduate School of Humanities and Social Sciences, and Network for Education and Research on Peace and Sustainability, Hiroshima University, Higashi-Hiroshima, 739-8530 Japan

## Abstract

**Background:**

The implementation of sustainability at universities means that they can also play a key role in the transition to a low carbon economy, and in assisting global efforts towards decarbonisation. Yet, not all of them have so far fully engaged in this area. This paper reviews the state of the art on trends in decarbonisation, and outlines the need for decarbonisation efforts at universities. It also reports on a survey aimed at ascertaining the extent to which universities in a sample of 40 countries across the various geographical regions are engaged in carbon reduction efforts, identifying the challenges faced.

**Results:**

The study has shown that the literature on the topic has been evolving over time and that increasing a given university’s energy supply from renewable energy sources has been the cornerstone of university-based climate action plans. The study also indicates that even though several universities are concerned with their carbon footprint and actively seeking ways to reduce it, there are some institutional obstacles that need to be overcome.

**Conclusions:**

A first conclusion which can be drawn is that efforts on decarbonisation are becoming more popular, with a special focus being placed on the use of renewable energy. Also, the study has shown that, from the range of efforts being made towards decarbonisation, many universities are setting up a team with carbon management responsibilities, have Carbon Management Policy Statements, and review them. The paper points towards some measures which may be deployed, so as to allow universities to take better advantage of the many opportunities an engagement in decarbonisation initiatives offers to them.

## Background

Decarbonisation refers to the reduction of carbon. In terms of the global society, it refers to the creation of economies and systems that reduce the amount of carbon produced or emitted in an environmentally sustainable manner [1]. The significant increase in carbon dioxide emissions has contributed to climate change over recent years [2]. A consequence of increased carbon emissions is the rise of the global average temperature which has been recorded at its highest over the past two decades [3]. The Paris Climate Agreement was signed by many governments with the aim of reducing greenhouse gas emissions. This is essential to mitigate the effects of climate change. Anthropogenic activities contribute largely to carbon emissions and thus it is important for decarbonisation practices to be put into place [4].

Furthermore, climate change has caused the occurrence of extreme weather events including flooding, droughts, heatwaves, wildfires, erratic storms, and the rise in sea levels [5–8]. This has caused much concern among people who fear that climate change will worsen in the future. Aside from this, climate change is promoting and enhancing the spread of many diseases which places stress on human health [9]. Therefore, it is imperative for systems to be created with carbon-free goals.

The major contributor to carbon emissions is the burning of fossil fuels to produce energy [2]. The energy sector needs to shift towards alternative and ‘clean’ sources of power to ensure that decarbonisation is realised. This can be achieved by using renewable energy sources such as wind, solar or hydropower [10].

European countries have taken decarbonisation policies seriously and have incorporated methods into ensuring that they will be “fossil-free” by 2050. Policies are designed to ensure that decarbonisation occurs in electricity, transportation, buildings, heating, and industrial activity. For instance, the transportation sector in most countries contributes to a large amount of carbon dioxide emissions. This is referred to as energy-related carbon emissions and significantly contributes to climate change. Therefore, vehicles need to be designed to run on alternative sources of energy such as natural gas or hydroelectric power to ensure decarbonisation of the transport sector [11, 12]. The many needs in this field are being addressed using renewable energy sources and clean energy efficient technologies and practices [13] across a wide range of institutions, including at universities, as described in the next section of this paper.

## Carbon reduction efforts at universities

Carbon management and reduction as a research topic have been explored in the higher education context in the past decades (e.g. [14, 15]). Following the approaches of ecological footprint analysis (EFA) and carbon footprint analysis (CFA) a number of Higher Education Institutions (HEIs) worldwide have been measuring their respective ecological footprints (EF) [16–18] and carbon footprint (CF) (e.g. [19–21]). The EF is broader in scope and approach, as it captures the impact, expressed in global hectares (gha), of several components, such as energy use; water use; waste; mobility; procurement; infrastructure; and food. The CF specifically sets focus on the amount of CO_2_ emissions, expressed in tons (t) from different components. Three types of emissions are calculated: (1) ISO scope 1 emissions (direct emissions of the HEI, e.g. heating of buildings); ISO scope 2 (indirect emissions resulting from energy use); ISO scope 3 (other indirect emissions, e.g. resulting from commuting, procurement). Measuring the CF of universities is complex, and studies have shown that it is not feasible to compare results of CFA between different HEIs, due to differences in calculation methods used; interpretations of certain emission types; and (intentionally or unintentionally) excluding certain components, especially scope 3 emissions, from the calculation [22, 23].

Assessing the CFs of HEIs has several benefits. Having an exemplary function in society, universities can showcase how their daily operations contribute to societal sustainability transition, as has been outlined in several studies, e.g.: societal role, outreach [24, 25]; whole-school approach, practise what you preach [22]; campus as living laboratories [26, 27]. It is clear that attention towards CF analysis and -reduction has educational and societal benefits. Regarding campus operations, CF assessments ideally also result in future strategic interventions to reduce carbon emissions, e.g. through improving the built environment or investing in renewable energy [22, 28]. In a worldwide study, HEIs reported taking measures to reduce energy use, improve energy efficiency, and encourage renewable energy, as well as measures related to mobility, such as carbon offsetting and behavioural change of students and employees [29].

The majority of published articles present either (1) case studies of individual HEIs on CFA and CF reduction (e.g. [17, 20–22]; and/or (2) carbon management in higher education context (e.g. [15, 30, 31]. As a concept and practice, “decarbonisation” is yet hardly discussed in the current academic body of knowledge. The explicit mentioning of the term in relation to HEIs is scarce, and few studies refer to the potential of decarbonisation technologies at HEI campuses [32–34]. Apart from contributing to environmental, educational, and societal benefits, implementing decarbonisation efforts is believed to provide economic benefits for HEIs as well, although this is currently still being debated in the literature [14]. With constantly changing legislative boundary conditions, it is often difficult for HEIs to measure economic benefits in the short- and long-term [32].

A growing number of HEIs worldwide are committing to becoming carbon-neutral. In order to reach this goal, a variety of decarbonisation pathways have been described in the literature, which individual HEIs can apply and combine, such as: reducing energy consumption; renewable energy projects; carbon offsets; improving energy efficiency; power purchase agreements; open-market renewable energy certificates [35]. While many of the case studies present best practices, success factors, and strategies to reduce carbon emissions and improve carbon management (e.g. [30, 36], the uncertainties and risks associated with (long-term) decarbonisation decisions, such as carbon lock-in, are insufficiently addressed. Carbon lock-in refers to the process in which dominant (fossil fuel) technologies are still preferred over low carbon alternatives. Worsham and Brecha [36, p.436] refer to three types of carbon lock-in, that are mutually reinforcing: (1) infrastructural and technological carbon lock-in; (2) institutional carbon lock-in; (3) behavioural carbon lock-in. A lack of focus on these carbon lock-ins and associated risks comprises the risk that “higher education institutions unconsciously make decisions now that will hinder their abilities to meet their climate goals” in the future.

The term ‘deep decarbonisation’ refers to decarbonisation efforts that drastically aim to reduce carbon emissions and ultimately reach carbon neutrality [32, 37]. From a national policies perspective, three pillars of deep decarbonisation have been defined: “(i) energy efficiency and conservation, including structural and behavioural changes; (ii) decarbonisation of energy carriers (electricity, heat, liquids, and gases); and (iii) end-use switching to these low-carbon carriers” [38, p. 263]. These three pillars are also relevant within the HEI context, especially those oriented towards energy efficiency and decarbonisation of energy carriers. In order to reach carbon neutrality in HEIs, centralised measures are needed, also referred to as critical energy infrastructure decisions, such as installing carbon-neutral heating installations [32].

(Deep) decarbonisation in higher education can be seen as a way forward to the transition towards low-carbon futures. The COVID-19 pandemic forced HEIs worldwide to shift to digital ways of teaching, which directly affected the daily campus operations, related to mobility (students and employees commuting to the campus) as well as energy use [19]. However, effects of hybrid, online, or distance education have been studied earlier in relation to the CF of higher education (e.g. [38–40], the COVID-19 pandemic has led to a growing understanding of how such modes of distance learning might contribute to envisioning low carbon futures.

## Methods

This study aimed to provide a greater understanding of the status of decarbonisation efforts that HEIs around the world contribute to by integrating climate solutions into their teaching, research, and operations activities, identifying the challenges they face within a 2050 perspective. To properly address the main objective of this research, a cross-sectional descriptive study was conducted, complemented by a survey. This type of dual study approach offers many advantages when it comes to shedding light on a given research topic, with the aim of fostering a greater understanding of new concepts or phenomena [41, 42].

The first step consisted of bibliometric analysis. Advances in text mining techniques have provided unprecedented opportunities to understand the overall structure and major focus areas of academic fields. For this purpose, different bibliometric analysis tools have been developed to identify influential sources, publications, and authors. Such tools can also be used to understand what thematic areas have received more attention. The latter is of interest to this study as it wants to find out what topics have received more attention in the literature related to university-based decarbonisation efforts. For this purpose, the study relies on the term co-occurrence analysis provided by VOSviewer, a frequently used bibliometric analysis technique [43]. The input data for term co-occurrence analysis are details of academic papers indexed in scientific databases. Here, the Web of Science (WoS), was used because of its broad coverage of peer-reviewed publications related to the study topic. To retrieve the relevant literature, a broad-based search was performed, which included a combination of different terms related to decarbonisation efforts at universities (see Appendix A). To develop the search string, an initial and simple combination of terms was used. However, after the initial search, it was also noticed that other terms, such as climate-neutral and carbon management, are also relevant and should be included. The final search was conducted on March 7, 2021, and returned 434 articles. The titles and abstracts of these articles were screened, and irrelevant papers were excluded. In the end, 116 papers were selected for final analysis using VOSviewer [44]. The output of term co-occurrence analysis is in a network of nodes and links, where node size is proportional to the term frequency and link width is proportional to the strength of the connection between two terms. Terms that are close to each other form thematic clusters that will be discussed in the “Results” section.

The second research method used in the second step was an online questionnaire. This was designed by a multidisciplinary team to be applied to students, researchers, educators, and administrative staff, to collect data regarding the decarbonisation efforts at HEIs. The questionnaire was designed based on previous literature which discusses decarbonisation issues [45–48].

The questionnaire, composed of 25 questions, was divided into 4 main parts: the first one aggregates 8 questions concerning participant background; the second part presents a set of 10 questions on the decarbonisation in campus operations to know if and how HEIs are performing actions and strategies; the third part, constituted of 4 questions, concerns decarbonisation in teaching and research and was designed to understand if this topic is incorporated in courses curriculum and teaching; the final part poses 3 questions to identify the main challenges and drives that HEIs are facing to implement decarbonisation strategies. The full questionnaire is presented in Appendix B. A pre-test was carried out by a group of academics whose fields of expertise lie within the scope of sustainable development research, to ensure that all relevant issues were considered and to check redundancies or similar items, as well as to evaluate the writing and sequence of questions. This process enables the questionnaire to be adjusted and redundant questions eliminated [49]. The language used was English.

The final version of the survey was administered through Google Forms and initially shared with scientific experts mailing lists and the network of the Inter-University Sustainable Development Research Programme (IUSDRP). A snowball sampling strategy was chosen to reach different viewpoints in a very small amount of time. Furthermore, in facing an emerging topic, such as decarbonisation, this sample allows us to reach the results faster and to provide up-to-date evidence [50]. The survey collected 110 responses between March and May 2021.

## Results and discussion

This section was divided into two main parts. The first, relying on the bibliometric analysis, while the second was devoted to analysing the data collected from the survey.

### Results of the bibliometric analysis

**Fig. 1:**
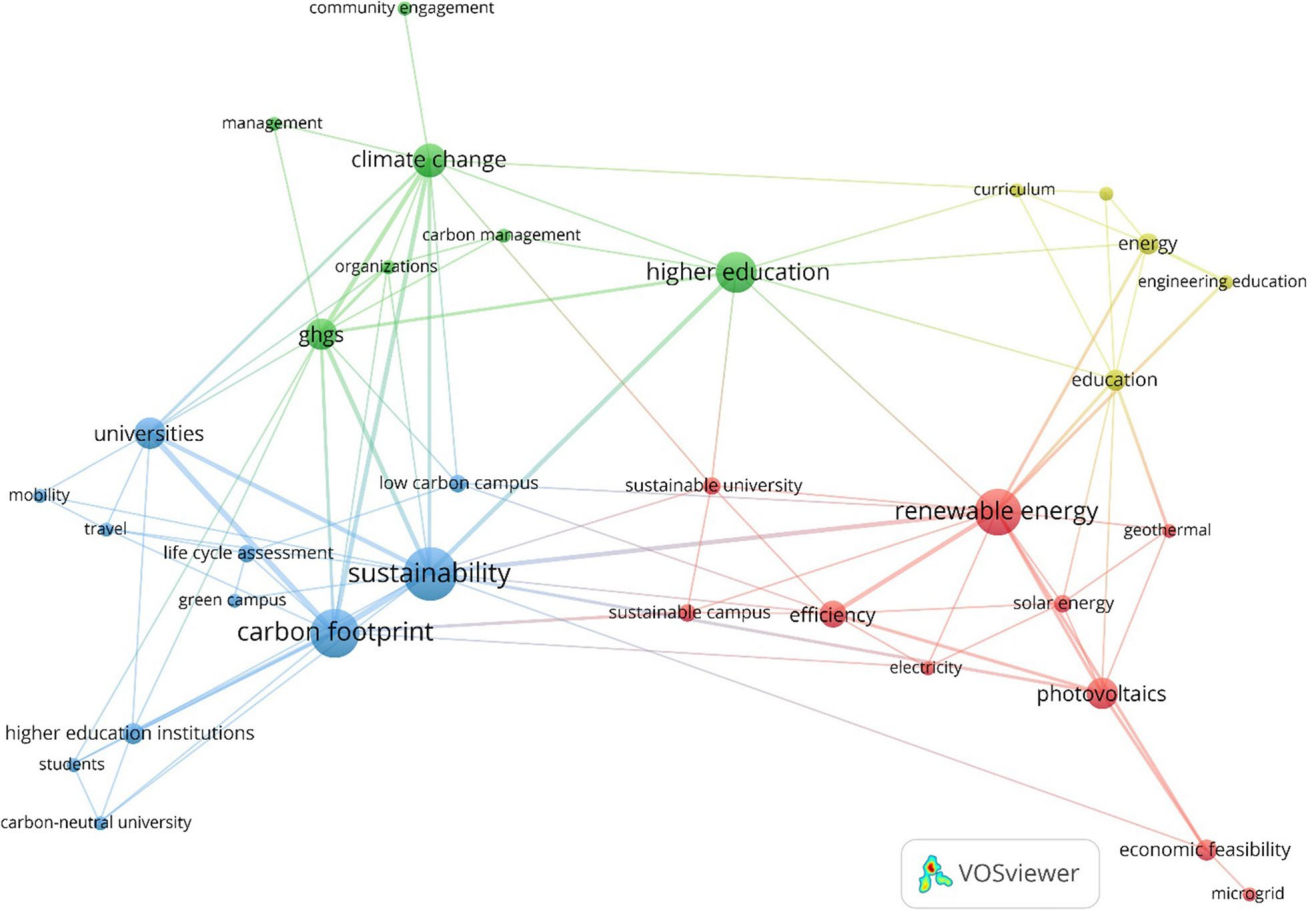
Output of the term co-occurrence analysis

Four different thematic areas can be identified from the output of the term co-occurrence analysis that is shown using different colours (Fig. 1). In the blue cluster, ‘carbon footprint’ and ‘sustainability’ are two dominant terms indicating that university-based decarbonisation efforts are closely related and/or part of broader efforts aimed at creating sustainable universities [51, 52]. Such decarbonisation efforts have been developed and practised under different initiatives such as low-carbon campus [16, 53], green campus [54], climate-neutral campus [52], and carbon-neutral university [55]. In these initiatives universities have functioned as living labs, practising a wide range of activities such as implementing innovative pilot projects [56], promoting sustainable behaviours among students [57], and examining the performance of universities as small-scale models of cities [58]. A common focus area of these campus-based efforts has been implementing renewable energy-based projects that are discussed under the red cluster. The blue cluster also includes terms ‘mobility’ and ‘travel’ that refers to issues related to reducing community- as well as long air travel-related emissions of students and faculty members [59, 60].

As mentioned above, increasing university energy supply from renewable energy sources has been the cornerstone of university-based climate action plans. This is evident in the red cluster, where terms such as ‘renewable energy’, and ‘photovoltaics’ are dominant. Obviously, of different renewable energy sources, solar energy has received more attention [61, 62]. The expansion of such renewable-energy-based initiatives is also expected to enhance efficiency by contributing to reducing energy loss in the distribution lines. It also facilitates the optimisation of energy management through, for instance, deploying microgrids that are integrated with renewable energy sources [63]. The economic feasibility of such systems has also been demonstrated through university-based pilot projects [64].

Energy-based initiatives in universities have also provided opportunities for enhancing student knowledge about renewable energy sources and technologies and this is also highlighted in the yellow cluster. Some universities have also integrated such subjects into the curriculum. This is believed to be important for further promotion and development of renewable energy technologies in the future [65]. Finally, the green cluster is dominated by terms such as ‘climate change’, and greenhouse gas (GHGs) that are linked with key terms related to management and governance. Clearly, effective management approaches are essential to ensure the success of university-based efforts towards decarbonisation [66]. Such management efforts should also be aware of the potential benefits of stakeholder engagement and community outreach for maximising performance through providing synergistic opportunities [67].

### Results of the survey

#### Demographics

**Fig. 2:**
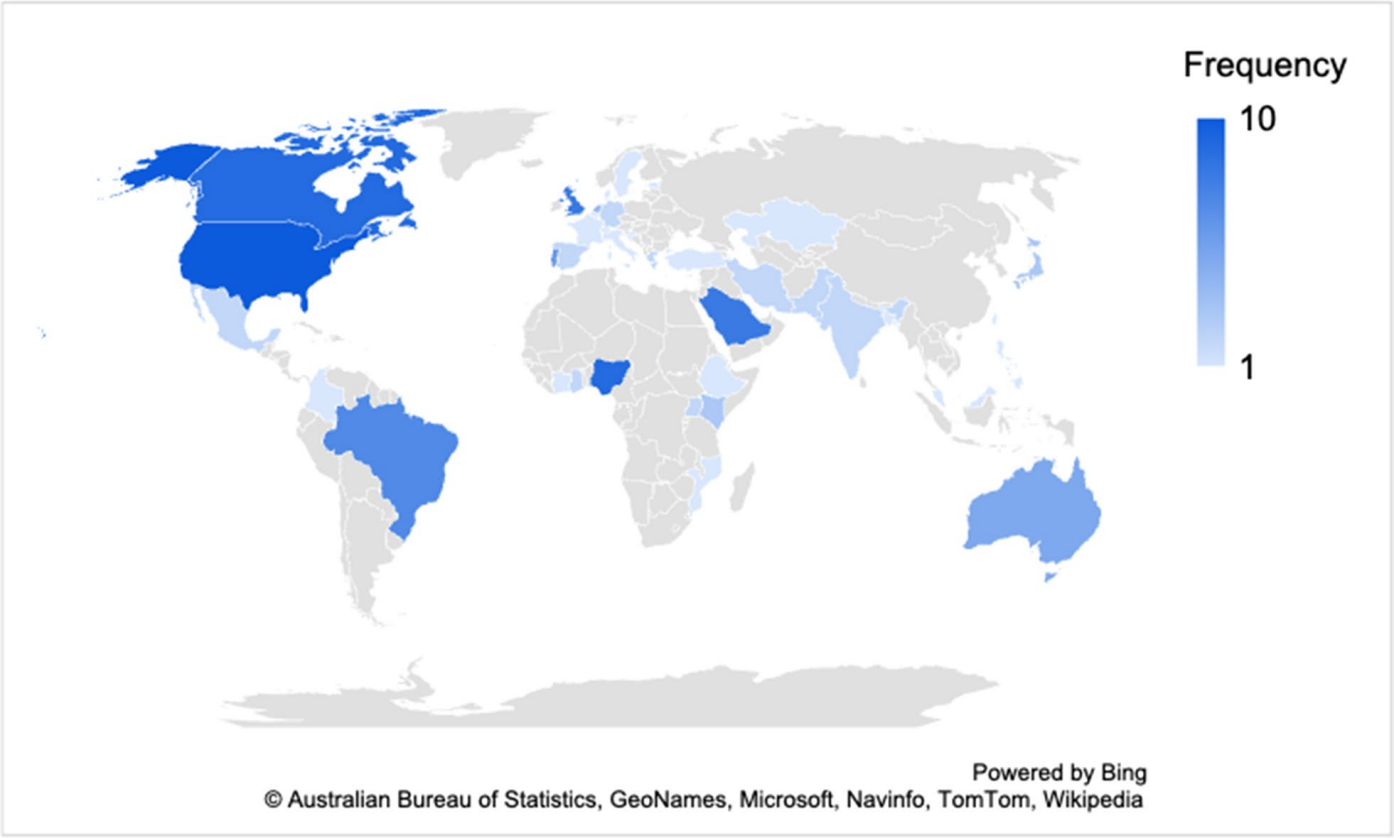
Country of the sampled respondents

The first part of the survey aimed to know the respondents' profiles. In total 110 responses were collected from 40 countries, as listed in descending order of a number of responses by country: United States (9.1%), Canada (8.2%), Nigeria (8.2%), Saudi Arabia (7.3%), United Kingdom (7.3%), Brazil (6.4%), Portugal (5.5%), Australia (4.5%), Netherlands (3.6%), Japan, Kenya, Germany, Ghana, Greece, India, Iran, Mexico, Pakistan, Spain, Uganda, Bangladesh, Belgium, Bosnia, Colombia, Cote d'Ivoire, Croatia, Denmark, Estonia, Ethiopia, France, Italy, Kazakhstan, Malaysia, Malta, Mozambique, Philippines, Sweden, Switzerland, Taiwan, and Turkey The most representative countries had the percentage of respondents assigned in parenthesis. A map of the countries where the sampled respondents live is shown in Fig. 2. The socio-demographic characteristics of the sample are summarised in Table 1.

**Table 1:** Socio-demographic characteristics of the respondents

Variable	Categories	Number (*n* = 110)	%
Region of your college/university	Africa	20	18.2
	Asia	23	20.9
	Europe	33	30.0
	North America	19	17.3
	South America	10	9.1
	Oceania	5	4.5
Gender	Male	68	61.8
	Female	37	33.6
	Other	1	0.9
	Prefer not to say	4	3.6
Age	18–32 yrs	15	13.6
	33–47	52	47.3
	48–62	35	31.8
	More than 63	8	7.3
University classification	Public	87	79.1
	Private	19	17.3
	Others (charity, philanthropic, foundation special status)	4	3.6
Highest education level	Secondary school or lower	1	0.9
	Tertiary education	10	9.1
	Postgraduate (e.g. MA/MSc, Ph. D.)	99	90.0
Primary position at the college/university	Lecturer/professor	52	47.3
	Researcher	17	15.5
	Student	12	10.9
	Administrative staff	15	13.6
	Higher management	11	10.0
	Sustainability education	1	0.9
	Researcher and lecturer	1	0.9
	Professor Emeritus	1	0.9
Main area of knowledge	Social sciences	41	37.3
	Engineering and technology	25	22.7
	Natural sciences	18	16.4
	Medical and health sciences	2	1.8
	Agricultural sciences	6	5.5
	Arts & humanities	5	4.5
	Sustainability and environmental studies	13	11.8

Prevailing in the sample are participants from Europe (30.0%) and Asia (20.9%), although the remaining continents are not imbalanced and there are no huge differences among them, allowing having an overview of the worldwide status of decarbonisation efforts at universities within a 2050 perspective. The majority are males (61.8%) and aged 33–47 years (47.3%), followed by those aged 48–62 years (31.8%). Regarding the university classification, public universities were more participative in the study (79.1%) as well as postgraduate participants (90.0%). Lectures/professors (47.3%) and researchers (15.5%) represent the vast majority of the participants. They are mainly working in the field of social sciences (37.3%) and engineering and technology (22.7%).

#### Decarbonisation in campus operations

The second part of the survey addressed the topic of decarbonisation in campus operations. As will be detailed here, the results obtained on “Decarbonisation in campus operations” is mainly focused on energy efficiency and consumption reduction, as shown in Box 1. It highlights the internal efforts most addressed in low carbon policy in universities and in Box 2 that indicates the topics most addressed in decarbonisation campus operations. The results in Table 2 suggest that universities seem to be making an effort towards implementing a carbon management structure in HEIs.

**Table 2:** Significant statements expressed by the respondents in relation to the carbon management structure adopted by the university

Most relevant responses	%
We have set up a team with carbon management responsibilities We have a clear Carbon Management Policy Statement We are set up to regularly review our carbon impact and revise our action plan We have a defined person with carbon management responsibilities We communicate the results of our carbon management progress to staff/customers We have carried out a one-off ‘Carbon Footprint & Recommendations’ process We currently have no structure in place for ensuring ongoing carbon management We are not doing anything When we are making decisions, we generally consider the carbon implications We have implemented a permanent carbon management system We are currently developing a carbon management programme	36.4 34.5 31.8 30.9 30.9 29.1 26.4 20.9 19.1 15.5 12.7

**Fig. 3:**
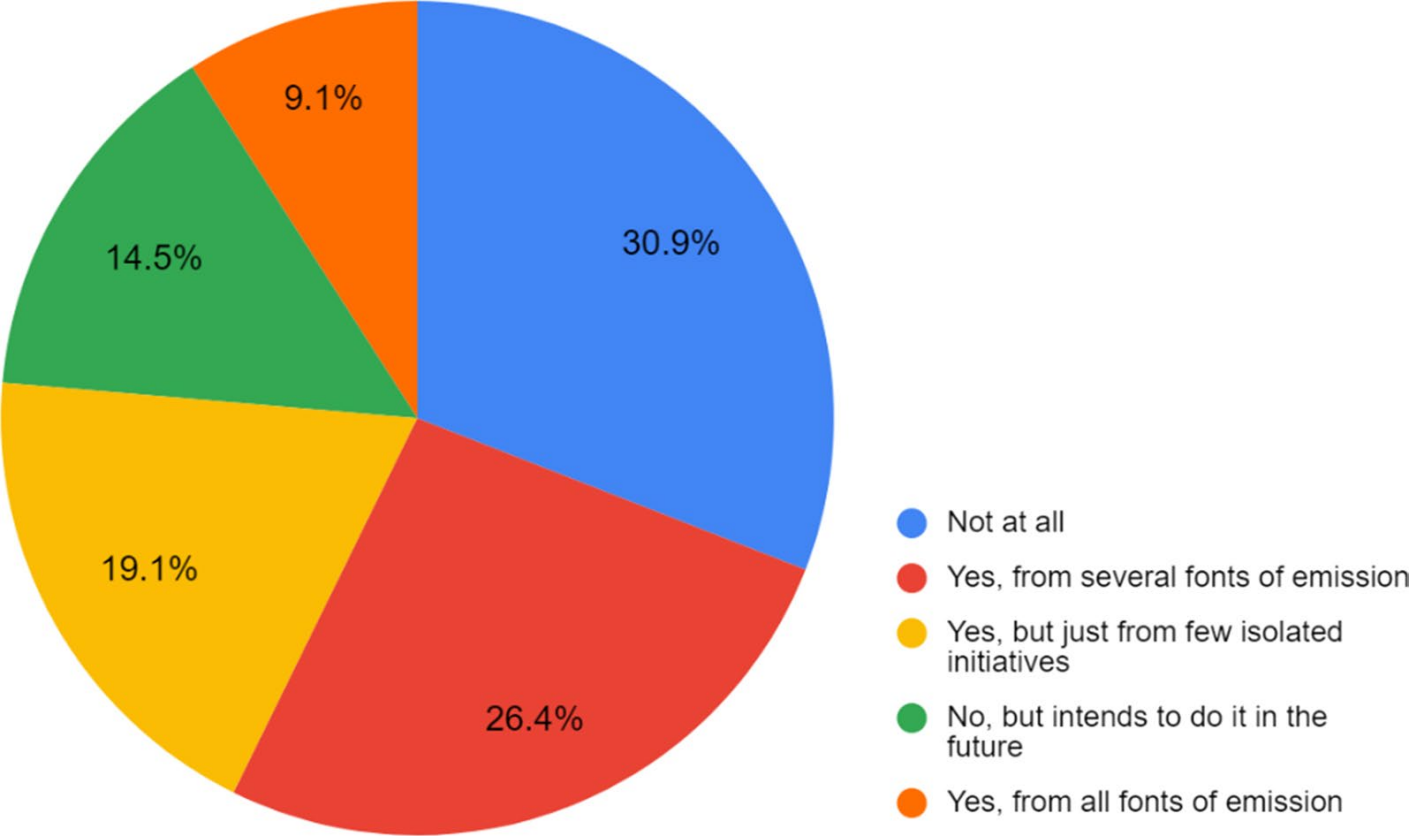
Assessing whether the university has a monitoring process to control emissions of greenhouse gases

**Fig. 4:**
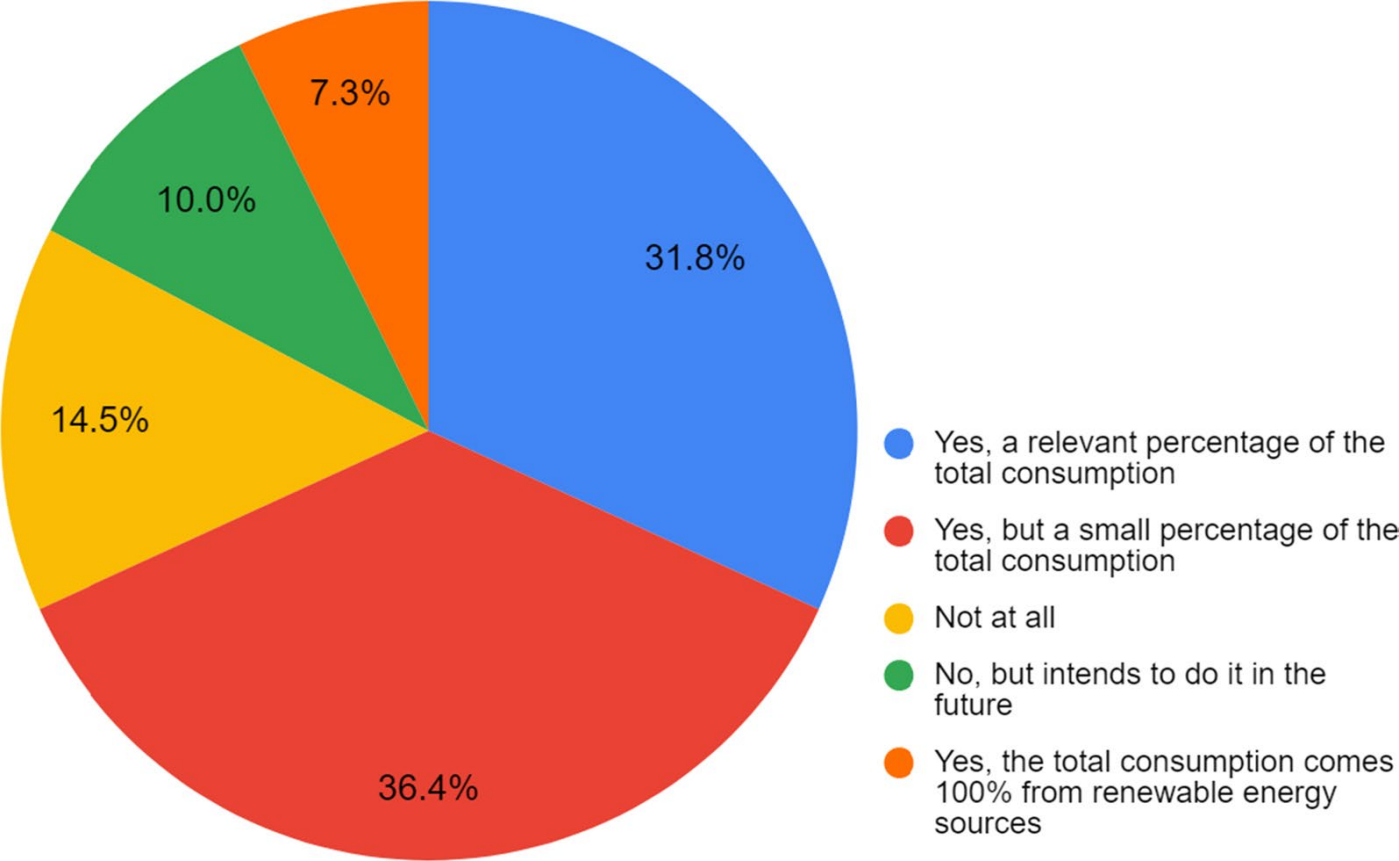
Assessing whether the university produces or purchase part of its energy from renewable energy sources

However, in practice, when addressing specific questions (Fig. 3), those efforts are not that clear. 36.4% of the respondents (Fig. 4) state that their universities do produce or purchase part of their energy from renewable sources, which is a big step in the shift direction. That same focus is observed when analysing the responses towards trends in decarbonisation in the future, with 77.3% pointing out the need to increase renewable energy (Table 3). Both now and in the future (Table 3), carbon emissions reduction is emphasised by many (75.5% now and 70.9% in future 2050).

**Table 3:** Most relevant statements expressed by the respondents about the important current trends and cross-cutting 2050 future expected trends involved in decarbonisation

Most relevant responses	Current trends (%)	Future expected trends (%)
Reduced carbon emissions	75.5	70.9
Increase the use of renewable energy	74.5	77.3
Promote energy efficiency	70.9	68.2
Increase social awareness and promote behaviour-change	67.3	66.4
More sustainable transportation	60.9	59.1
Energy and carbon literacy	53.6	57.3
Climate policy instruments	51.8	67.3
Levels of energy demand	49.1	50.0
Social or environmental justice	46.4	58.2
Technology support	39.1	–
Energy crisis, price, and policy	29.1	45.5

Regarding the type of sources used, 75.8% of respondents indicated solar energy as the primary source in response to the question regarding the most used sources to produce energy, followed by wind energy (28.6%), hydropower (25.3%), thermal energy (biomass) (12.1%) and geothermal energy (9.9%), with other sources being reported by a few cases.

In relation to the internal efforts pointed out to achieve progress in low carbon policy within the university, respondents gave a diversity of responses. However, the focus can be considered as pointing out to energy consumption reduction and improved efficiency, also highlighting the role of renewable energy and campus sustainability, as it is highlighted in Box 1.

**Figure:**
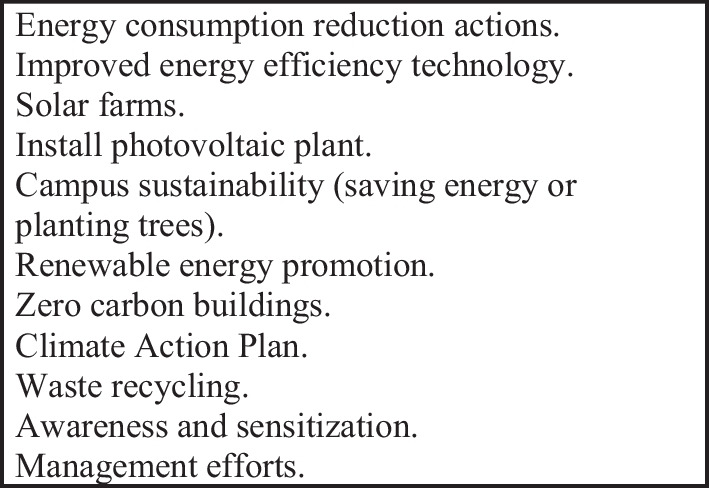
Box 1. Topics highlighting the internal efforts most addressed in low carbon policy in universities

While these results are in line with a previous global review on sustainability integration in higher education (Lozano et al., 2015), it suggests that HEIs are struggling to take concrete measures towards carbon neutrality. However, some universities in our sample seem to have ‘no structure in place for ensuring ongoing carbon management’ (26.4%) or are ‘not doing anything’ (20.9%) as described in Table 2. Having only partially, or even not shifted to renewable sources, exemplifies that (deep) decarbonisation efforts are difficult to realise at universities. This might be under the influence of institutional carbon lock-ins (cf. [35]).

In Table 3 is shown in the second column the trends of current topics and in the third one the trends of expected future topics that were considered more relevant to decarbonisation issues at HEIs.

In 7 out of the 10 trends most reported by respondents, it is shown that there is no expressive difference between what is currently expected and what will be expected in the future, demonstrating the perception of urgency in addressing issues related to climate change by HEIs. It suggests the path that HEIs might follow to effectively contribute to achieving on-time what was established in the Paris Agreement. The results are in line with the work of Leal Filho et al. [68], which aimed at identifying the extent to which matters related to climate change are addressed within the teaching and research practices at universities, with a focus on the training needs of teaching staff.

It is also noticed that statements more related to concrete aspects of campus operation that are usually in the scope of each HEI's internal decision-making, such as “Reduced carbon emissions”, “Increase the use of renewable energy” or “Promote energy efficiency”, were referred to more frequently by the sample of respondents. It may indicate that in the perception of them HEIs have been focused on carrying out actions of this type. In contrast, “Energy crisis, price and policy”, “Social or environmental justice” and “Levels of energy demand” were marked by a smaller number of respondents possibly because they may perceive them as topics that are in the sphere of responsibility of governments and multilateral structures and therefore HEIs have little or no influence on those issues.

EU has embraced Europe to be a climate-neutral continent by 2050 [69]. That can only be achieved through the participatory involvement of all stakeholders and universities are crucial in this process, demanding further collaborative efforts in working together with industrial R&D departments [47, 69]. More than a set of goals, the energy transition journey in Europe will involve designing specific policies and measures [45]. Enquired whether the university had partnerships to design/implement a carbon-neutral plan, 44.5% of the respondents stated no partnership was developed with other stakeholders, followed by 24.5% of respondents that mentioned both city hall and companies/industry partnerships. NGOs were mentioned by 16.4% of the respondents and civil society groups by 13.6%. Financial institutions corresponded to 6.4% and insurance companies to 3.6%. Universities and other organisations were mentioned by less than 1%. 1.8% of the respondents clearly mentioned no partnership at all.

Asked for additional comments on decarbonisation campus operations, some specific concerns were mentioned. The more reported comments are listed in Box 2.

**Figure:**
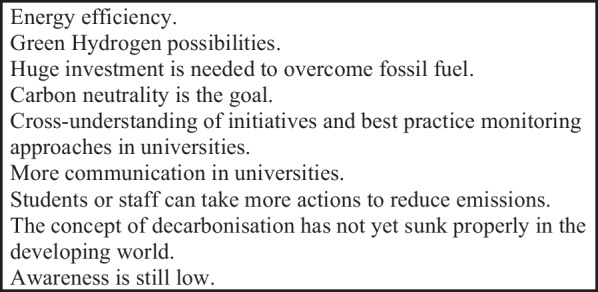
Box 2. Topics most addressed in decarbonisation campus operations

Fossil-based energy is the main contributor to climate change, responsible for around 60% of global GHG emissions [70]. Countries intention to be virtually "fossil free" by 2050 [13]. Thus, efforts towards decarbonisation demand a holistic approach. In that respect, universities are considered to be a driving force in change, contributing to infrastructure development, technology improvement for cleaner and efficient energy globally, stimulating growth, and protecting the environment. In order to attain SDG 7 (affordable and clean energy) objectives to decarbonise the global energy system, all stakeholders are called to intervene. At the same time, decarbonising the energy system also impacts other SDGs, such as those addressing air pollution (SDGs 3 and 7), clean water (SDG 6), and food security (SDG 2) [71]. Low carbon economic strategies investment will support economic development in the future [72, 73].

The transport sector, representing a decarbonisation challenge due to carbon-intensive oil products used [74], accounts for more than 50% of total oil consumption [75] and universities seem to find it a relevant pathway towards decarbonisation efforts. Table 3 shows that sustainable transport is reported by almost 2/3 of the respondents (60.9%). Referring back to the EF literature, carbon emissions related to transport and mobility constitute a major part of the total emissions, yet are difficult to tackle from the organisational perspective [22]. For example, changing policies for internationalisation directly influences the research activities of faculty staff and might provoke resistance to such measures. A turn-away path in the political arena can be accomplished through fuel taxation (carbon tax/higher fuel prices) and emission standards, aiming to pursue energy efficiency and low emission.

Energy efficiency plays a major role in (deep) decarbonisation efforts [37]. While energy efficiency can be faced as the minimum energy to supply the same level of energy, the ecosystem-related approach goes one step further, aiming to safeguard the integrity of the ecosystem while simultaneously providing optimum energy services. Both views will ultimately aim to contribute to limiting human (GHG) emissions [70]. Scientists and researchers from universities are seen as reliable stakeholders in the energy efficiency field [48]. Accordingly, it is natural that this topic is considered to be important in the reported responses found in the analysis of the results (Box 1, Box 2 and Table 4), reflecting the fact that universities see energy efficiency efforts as a way to tackle 2050 decarbonisation efforts. The same exercise can be applied to the “Zero carbon buildings” goal, pointed out by the respondents (Box 1) in the scope of the efforts needed to be addressed by universities to achieve a low carbon policy. Global carbon targets cannot be attained without considering the buildings and universities represent that respect significant contributors, particularly those involving the technology areas, with experiments being carried out continuously, demanding additional energy effort. Clearly, energy topics addressed in the survey in the university context are all related to the sustainable pathway involved in efforts to progress towards a low carbon future. According to Donkor and Mearns [70], societies embracing sustainability will ultimately be the most successful and prosperous of the future and universities in particular, through research and also due to the important role played as stakeholders, are important players.

**Table 4:** Perception of the respondents regarding the integration of decarbonisation into teaching and research and its potential to engage students

	(a) Decarbonisation integrated into teaching (%)	(b) Decarbonisation integrated into research (%)	(c) Potential of decarbonisation themes to engage students (%)
Not at all	5	9	3
To a small extent	26	25	5
To some extent	40	34	25
To a moderate extent	19	16	27
To a great extent	9	16	40

Waste recycling is mentioned in Box 1 as a topic to be addressed in the scope of low carbon policies and internal efforts. Low costs technology to control emissions from waste and wastewater handling and fossil fuel production and use are available through waste recycling and wastewater treatment plants [76] and further efforts in developing countries, those with the poorest management systems will significantly impact the reduction of global anthropogenic emissions in the 2050 timeframe, particularly methane.

Policy instruments are mentioned by 67.3% (Table 4) as important cross-cutting issues in relation to 2050 future trends in decarbonisation. However, they are unlikely to be realised without strong policy incentives, as advocated by Zhou et al. [77] when investigating low carbon investment needs involved in climate policy scenarios and we do know that universities tend to neglect specific aspects that could further be enhanced at this respect.

Education is essential in developing climate literacy at all levels of study and across all disciplines [78]. Communication, on the other hand, is highlighted in Box 2 as essential to decarbonisation efforts. Communication is essential [79] in the context of universities’ actions to tackle decarbonisation efforts. The survey shows that energy and carbon literacy (53.6% in Table 4) in the context of universities represents an important trend in the decarbonisation topic, in agreement with what happens in the household context [48]. Awareness and sensitisation towards promoting behaviour change are important and highlighted through the results obtained in the survey, but it will also be crucial to persuade collective decision-making stakeholders to pursue that awareness, interest, and enthusiasm at the collective level [45]. Driving changes in behaviour could shape consumption habits, motivating further changes to emerge [46].

Biresselioglu et al. [45] found that barriers to European decarbonisation are frequently described as being confined to lower-level collective decision-making context, while the transition to low carbon is deemed inevitable for higher levels of governance, resulting in a strong will to overcome those barriers. The efforts to achieve decarbonisation at universities reported in this study seem to indicate that universities aim to play a relevant role in this respect. Several of the organisations which took part in this study seem to be interested in achieving campus sustainability, acting as living labs.

#### Decarbonisation in teaching and research/challenges and drivers

Table 4 presents the distribution of the sampled respondents in questions on how they perceive the integration of decarbonisation into teaching (a) and research (b), as well as the potential of decarbonisation themes to encourage students to be able to contribute to low carbon efforts at their institutions (c). It can be observed that factual elements (a, b) returned fewer positive responses, with more than half of the sample distributed among the mid-low response options (small/some extent). On the other hand, the normative element—potential to contribute—had over 90% of the sample distributed among the top three options (great/moderate/some extent). This finding indicates how the topic is perceived as important to mobilise students, even though it might have not been applied so satisfactorily in teaching and research.

After this set of questions, respondents were given an open space to comment on their views of decarbonisation in teaching and research. Table 5 presents these comments, classifying them among references to challenges, supporting arguments, and positive stories.

**Table 5:** Open-space comments of the sample around the topic of decarbonisation in teaching and research

Main topic	Quotes from respondents
Challenges	“Decarbonization remains predominantly technology focused, there is a need to better integrate social sciences and also to consider decarbonization from a systems level perspective versus one-off technologies” “Not enough courses are related to carbon emission reductions in the social science field”
	“Here, decarbonization is usually encountered as a theoretical issue in climate change research.” “Too much curriculum is focused on all sources (including maintaining and making fossil more efficient), not picking & choosing future energy source winners. That doesn't prepare students to be leaders though.”
	“It can and should be much more, but many teachers are not interested in the theme, researchers much more” “The curriculum is being 'greened' including around carbon emissions and climate change, but it is fragmented and not well coordinated.”
Supporting arguments	“It is one concept that is gathering international attention, both in policymaking and academia, and worth embedding in teaching and research.” “New research projects aligned to decarbonization are needed. An increase in inclusion, in curricular programmes” “The decarbonisation should be included in the curricula of the Universities” “We need more affirmative action” “We need to learn more about this” “We should include it in all study plans” “It's important to teach”
Positive stories	“I think many of our students come to us highly motivated about decarbonisation and other environmental protection. They fit with what many of our staff want to teach and research. Our campus has a high fraction of research and teaching concerned with renewables, ecology, geography, and sustainability.”
	“Significant strengths” (e.g. centres/institutes on climate change/sustainable energy) “We are developing a new model on this topic” “We are launching a major effort” (referred to as a partnership for a centre on carbon capture)
	“We have an active focus on using campus operations as a classroom for students to learn about decarbonization” “We have embedded this in our teaching and research and more needs to be done across the schools” “We introduce the topic of decarbonization in our education in some of the courses, such as the one focusing on Closed Loop Supply Chains. Students have the option to choose for a master thesis topic on ‘footprinting’, which includes carbon or ecological footprinting.” “Within our new plan is the target for all programmes at each level (first year, second year, etc.) to include the climate & ecological crisis in their indicative content”

**Fig. 5:**
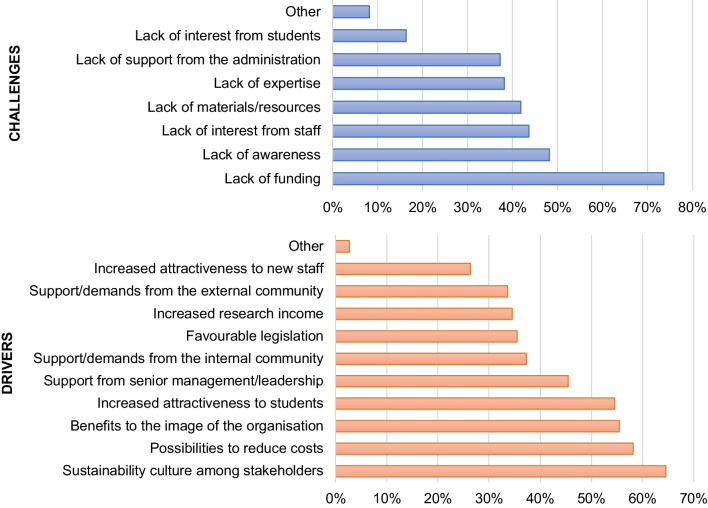
Challenges and drivers for the implementation of decarbonisation initiatives at the sampled universities

This study was also interested to investigate which are the main challenges and drivers for the implementation of decarbonisation initiatives at HEIs. The results in Fig. 5 are presented as the percentage of respondents who indicated these aspects. When challenges are concerned, three in four respondents have indicated lack of funding as an important aspect to be considered. Lack of awareness of interest from staff and of material/resources are also among the most selected challenges (between 42 and 48%), reinforcing the need for effective management approaches [66]. On the other hand, the least voted challenge was lack of interest from students, indicated by only 16% of the sample. Other listed challenges include lack of governmental policies/support, problems in prioritisation, and distribution of funding.

As for drivers, four aspects were indicated by over 50% of the respondents: increased attractiveness to students (55%), benefits to the image of the organisation (55%), possibilities to reduce costs (58%), and sustainability culture among stakeholders (65%). The increased attractiveness to students can refer to the sustainability and climate action appeal, but also to the availability of on-campus initiatives to promote better learning opportunities and behaviour-change [26, 29]. Although controversial, the possibility of investments in decarbonisation for economic benefits is discussed in the literature [14] and the survey results confirmed this as an important supporting argument. A few other examples of drivers were presented: interest of professors, researchers, and students, their active work in the area, and plan/policies from the external community (e.g. when a local council has a carbon zero plan).

It should be pointed out that there is a risk that some respondents may not be fully aware of the efforts being made at their universities to decarbonise. Despite this and considering the findings of this study, efforts should be expanded to increase the institutionalisation of sustainable development initiatives aimed at promoting decarbonisation in HEIs [80]. This may take place by developing clear policy statements, and carbon management systems. The institutionalisation and formalisation of action plans are considered in the literature as challenges that HEIs must overcome in order to adequately assume their role in building more sustainable societies [81, 82].

In this context, the engagement of the academic community is crucial in implementing sustainable development at HEIs and internalising it into the institutional culture [26, 83].

## Conclusions

This article reports on a study focusing on decarbonisation at HEIs, which consisted of bibliometric analysis and an online survey taking place. A first conclusion which can be made is that efforts on decarbonisation are becoming more popular, with a special focus being placed on the use of renewable energy. Also, the study has shown that, from the range of efforts being made towards decarbonisation, many universities are setting up a team with carbon management responsibilities, have Carbon Management Policy Statements, and review them. Furthermore, over 75% of the respondents indicated solar energy as a primary source in their institutions. A reduction in carbon emissions is seen in most cases as a priority now and in the future, by a significant number of participants HEIs.

This study has some limitations. Firstly, it relied on the term co-occurrence analysis provided by VOSviewer. Even though this is a frequently used bibliometric analysis technique, it has a constraint in respect of the number of terms that can be assessed. Secondly, the online questionnaire survey was undertaken over a short period of time and could only involve HEIs staff who were motivated—and interested to take part in it. Thirdly, the size of the sample, which involved 110 respondents, cannot be regarded as representative, although relating 40 countries.

Despite these limitations, the paper makes a timely contribution to the literature in the sense that it provides a profile of the extent to which universities are engaging in decarbonisation efforts. Also, the sample, which entails respondents from 40 countries, illustrates the levels of emphasis the topic is currently having. The sample also fulfils the purpose of outlining some of the challenges that HEIs currently face in reducing their CF.

Some of the measures which may be deployed, so as to allow universities to take better advantage of the many opportunities an engagement in decarbonisation initiatives offers to them are:

- to promote wider awareness of the fact that decarbonisation is not a sole task for the university administration, but that it is a goal also to be embraced by staff and students;
- themes associated with decarbonisation such as CO_2_ emissions or the use of renewable energy, may be used as part of a wide range of courses, raising the awareness of students across the social and natural sciences, and not only in engineering, as it is sometimes the case;
- efforts to foster decarbonisation may be supported by the use of digitalisation, with tools that enable more resource and energy efficiency, and better network utilisation and new technologies that may contribute to climate protection;
- research at the interfaces of decarbonisation and overall climate protection efforts should be promoted more strongly.

In addition, bearing in mind the high demand for skilled workers in the field of climate protection, knowledge of matters related to decarbonisation may be helpful in meeting with the shortages of personnel in the labour market. This trend, in turn, gives an opportunity for universities to strengthen their further education programmes. Overall, in order to better tap the potentials of decarbonisation, greater emphasis should be given to giving universities incentives to better integrate decarbonisation as part of their transformation process.

## Data Availability

The datasets used and analysed during the current study are available from the corresponding author on reasonable request.

## Appendix A: Search string

(TS= (( “carbon neutral*” OR "climate action" OR "climate change in the curriculum" OR "climate mitigation" OR "climate change mitigation" OR “decarboni*” OR "carbon literacy" OR “renewable energy*” OR "zero carbon" OR "low carbon" OR "zero-carbon" OR "low-carbon" OR "net zero" OR "climate neutral" OR "climate-neutral" OR "NZEB" OR "carbon management" OR "carbon efficiency" OR “carbon footprint”) NEAR/15 (“universit*” OR “higher education institut*”) )) *AND ***LANGUAGE:** (English) 

*Indexes=SCI-EXPANDED, SSCI, A&HCI, ESCI Timespan=1900-2021*

## Appendix B: Survey

1. Country of your college/university:

____________

4. Gender

( ) Female, ( ) Male, ( ) Other, ( )Prefer not to say.

5. Age, (numerical – e.g., 32):

____________

6. Highest Educational Level:

( ) Secondary school or lower; ( ) Tertiary education; ( ) Postgraduate (e.g., MA/MSc, Ph. D.)

7. Primary position at the college/university:

( ) Student; ( ) Researcher; ( ) Lecturer/Professor; ( ) Administrative Staff; ( ) Higher management; ( ) Other______________

8. Which is your main area of knowledge:

( ) Natural Sciences; ( ) Engineering and technology; ( ) Medical and Health sciences; ( ) Agricultural sciences; ( ) Social sciences; ( ) Arts & Humanities; ( ) Other: _______

9. At the best of your knowledge, please mark the statements related to the carbon management structure adopted by your college/university.

( ) We have a clear Carbon Management Policy Statement; ( ) We have a defined person with carbon management responsibilities; ( ) We have set up a team with carbon management responsibilities; ( ) We have carried out a one-off ‘Carbon Footprint & Recommendations’ process; ( ) We are set up to regularly review our carbon impact & revise our Action Plan; ( ) We communicate the results of our carbon management progress to staff/customers; ( ) We have implemented a permanent Carbon Management System; ( ) We currently have no structure in place for ensuring ongoing carbon management; ( ) We are currently developing a Carbon Management programme; ( ) When we are making decisions we generally consider the carbon implications; ( ) We are not doing anything; ( ) Other: ____________

10. Does your university have a monitoring process to control its emissions of greenhouse gases?

( ) Not at all; ( )No, but intends to do it in the future; ( ) Yes, but just from few isolated initiatives; ( ) Yes, from several fonts of emission; ( )Yes, from all fonts of emission.

11. Does your university produce/purchase part of its energy from renewable energy sources?

( ) Not at all; ( )No, but intends to do it in the future; ( )Yes, but a small percentage of the total consumption; ( )Yes, a relevant percentage of the total consumption; ( )Yes, the total consumption comes 100% from renewable energy sources.

11.1 If so, which sources are used? (Multiple answer possible)

( )Hydropower; ( ) Solar energy; ( ) Wind energy; ( )Thermal energy (biomass); ( ) Geothermal energy; ( )Other: _____________

12. Which internal efforts can you point out within your college/university context to achieve progress in low carbon policy? ____________________________________________________________

13. In your opinion, important current trends in decarbonisation involve which of the following topics? (Multiple choices possible)

( ) Levels of energy demand Increase use of renewable energy; ( )Promote energy efficiency; ( ) Reduced carbon emissions; ( )Energy crisis, price, and policy; ( )Climate policy instruments; ( ) Increase social awareness and promote behaviour-change; ( )Energy and carbon literacy; ( ) More sustainable transportation; ( ) Technology support; ( ) Social or environmental justice; Other:___________

14. In your opinion, 2050 future expected trends in decarbonisation involve which of the following cross-cutting issues? (Multiple choices possible)

( ) Levels of energy demand; ( ) Increase use of renewable energy; ( ) Promote energy efficiency; ( ) Reduced carbon emissions; ( ) Energy crisis, price and policy; ( ) Climate policy instruments; ( ) Increase social awareness and promote behaviour-change; ( ) Energy and carbon literacy; ( ) More sustainable transportation; ( ) Social or environmental justice; Other: _____

15. Has your college/university partnered with other stakeholders to design/implement their carbon neutral plan? If so, with which partners? (Multiple choices possible)

( )It has not developed the plan/partnered with other stakeholders; ( ) City Hall; ( ) Insurance companies; ( ) Companies/Industry; ( ) Financial institutions; ( ) Civil society groups; ( ) NGOs; Other: _______________

16. Please, use this space If you would like to make any comments regarding decarbonisation in campus operations

______________________

17. To the best of your knowledge, is the topic of decarbonisation integrated into any teaching programs at your college/university?

( ) Not at all; ( ) To a small extent; ( ) To some extent; ( ) To a moderate extent; ( ) To a great extent

18. To the best of your knowledge, are your college/university involved in researching decarbonisation?

( ) Not at all; ( ) To a small extent; ( ) To some extent; ( ) To a moderate extent; ( ) To a great extent

19. Do you believe that decarbonisation themes have the potential to encourage students to be able to contribute to low carbon efforts at your college/university?

( ) Not at all; ( ) To a small extent; ( ) To some extent; ( ) To a moderate extent; ( ) To a great extent

20. Please, use this space If you would like to make any comments regarding decarbonisation in teaching and research

Challenges and drivers

21. Which elements pose a challenge to the efforts of implementing decarbonisation initiatives at your college/university? (Multiple answers possible)

( )Lack of expertise; ( ) Lack of awareness; ( ) Lack of interest from staff; ( ) Lack of interest from students; ( ) Lack of materials/resources; ( ) Lack of support from the administration; ( ) Lack of funding; Other: _________

22. Which elements represent drivers for the implementation of decarbonisation initiatives at your college/university? (Multiple answers possible)

( ) Sustainability culture among stakeholders; ( ) Favourable legislation; ( ) Benefits to the image of the organization; ( ) Possibilities to reduce costs; ( ) Increased attractiveness to students; ( ) Increased attractiveness to new staff; ( ) Increased research income; ( ) Support from senior management/leadership; ( ) Support/demands from the internal community; ( ) Support/demands from the external community; Other: _________

23. If you have specific examples of a case study or project involving decarbonisation at your university, briefly describe the experience (objective, method, impact, and results, for example).
